# Large-Scale Fabrication of Porous Gold Nanowires via Laser Interference Lithography and Dealloying of Gold–Silver Nano-Alloys

**DOI:** 10.3390/mi8060168

**Published:** 2017-05-24

**Authors:** Adrien Chauvin, Nicolas Stephant, Ke Du, Junjun Ding, Ishan Wathuthanthri, Chang-Hwan Choi, Pierre-Yves Tessier, Abdel-Aziz El Mel

**Affiliations:** 1 Institut des Matériaux Jean Rouxel, IMN, Université de Nantes, CNRS, 2 rue de la Houssinière B.P. 32229, 44322 Nantes cedex 3, France; Adrien.chauvin@cnrs-imn.fr (A.C.); Nicolas.stephant@univ-nantes.fr (N.S.); pierre-yves.tessier@cnrs-imn.fr (P.-Y.T.); 2 Department of Mechanical Engineering, Stevens Institute of Technology, Castle Point on Hudson, Hoboken, NJ 07030, USA; jding1@stevens.edu (J.D.); Ishan.Wathuthanthri@ngc.com (I.W.); cchoi@stevens.edu (C.-H.C.); 3 Department of Chemistry, University of California, Berkeley, CA 94720, USA; kdu1@berkeley.edu; 4 Northrop Grumman Mission Systems, Advanced Technology Laboratories, Linthicum, MD 21090, USA

**Keywords:** nanopatterning, nanoporous gold, laser interference lithography, dealloying

## Abstract

In this work, we report on an efficient approach to fabricating large-area and uniform planar arrays of highly ordered nanoporous gold nanowires. The approach consists in dealloying Au–Ag alloy nanowires in concentrated nitric acid. The Au–Ag alloy nanowires were obtained by thermal annealing at 800 °C for 2 h of Au/Ag stacked nanoribbons prepared by subsequent evaporation of silver and gold through a nanograted photoresist layer serving as a mask for a lift-off process. Laser interference lithography was employed for the nanopatterning of the photoresist layer to create the large-area nanostructured mask. The result shows that for a low Au-to-Ag ratio of 1, the nanowires tend to cracks during the dealloying due to the internal residual stress generated during the dealloying process, whereas the increase of the Au-to-Ag ratio to 3 can overcome the drawback and successfully leads to the obtainment of an array of highly ordered nanoporous gold nanowires. Nanoporous gold nanowires with such well-regulated organization on a wafer-scale planar substrate are of great significance in many applications including sensors and actuators.

## 1. Introduction

Nowadays, tremendous efforts have been dedicated to the development of scalable and efficient fabrication approaches of nanoporous materials since they exhibit exceptional properties, allowing them to be used in a large spectrum of applications including catalysis [1,2], sensors [3,4], and actuators [5,6]. Several methods have been explored to create this kind of nanoscale architectures including templating [7,8], galvanic replacement [9,10], oxidation [11], and dealloying [12,13,14]. The most common technique used to fabricate nanoporous metals is dealloying; it is to remove a less noble metal from an alloy in order to leave behind a highly porous skeleton of the noble metal [15,16,17]. Two phenomena take place during the dealloying process including (i) dissolution of the less noble metal and (ii) surface diffusion of the noble metal, which proceeds simultaneously at the leaching interface [18,19,20]. The dealloying process can be applied either by “free corrosion” in simple immersion of the alloy in a highly corrosive medium or by electrochemistry in diluted acid media. Compared to electrochemical leaching, free corrosion is much simpler as it allows for the creation of nanoporous materials conveniently at a relatively low cost [21].

Most studies on the fabrication of nanoporous materials reported in the literature involve dealloying thin films [22]. However, the fabrication of the nanoporous materials in one-dimensional arrays of nanowires improves the functional properties of the material in comparison to thin films due to the very high aspect ratio of such one-dimensional structures that allows enhancing their active specific surface area [23,24,25]. In comparison to nanoparticles, creating organized arrays of nanoporous nanowires over a large substrate area enables a simple integration of such functional materials in micro/nano-devices due to the simplicity in the manipulation [26]. When creating nanoporous nanowires, the main drawbacks are the short length and the poor organization of the structures after dealloying [27,28]. Recently, we have reported on a new approach to creating highly ordered and ultra-long planar arrays of nanoporous gold nanowires, combining wafer-scale laser interference lithography and a dealloying of gold–copper (Au–Cu) nano-alloys [29,30]. In brief, the process consists of depositing Au–Cu alloys by magnetron co-sputtering on a nanopatterned substrate by laser interference lithography, in order to form the Au–Cu alloy nanowires by the shadowing effect [31]. In the study, we investigate the effectiveness of using a lift-off technique instead of using the shadow effect. The dealloying process is applied to a planar array of gold–silver (Au–Ag) alloy nanowires prepared by Au–Ag stacked nanoribbons annealed at 800 °C for 2 h. Using free corrosion in concentrated nitric acid, we show how the nanoporosity can be generated within the nanowires. In particular, the influence of the Au-to-Ag ratio on the final organization and porosity of the nanowire is investigated.

## 2. Fabrication Scheme

**Figure 1:**
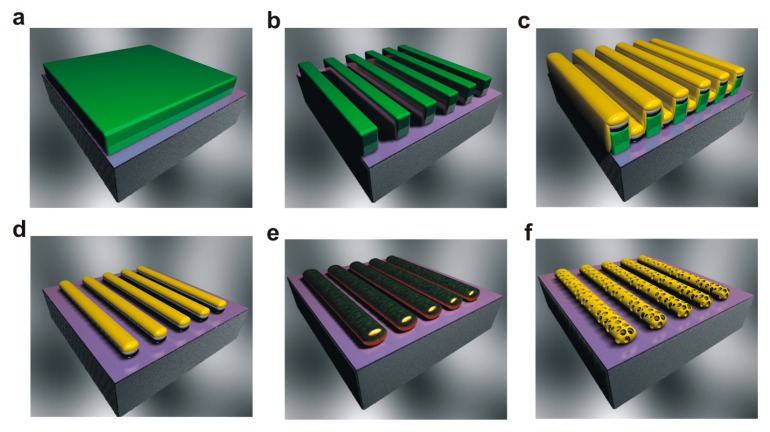
Schematic illustration presenting the fabrication procedure of nanoporous gold nanowires. (**a**) Deposition of 200 nm photoresist layer by spin-coating on a silicon substrate. (**b**) Formation of photoresist nanogrates by laser interference lithography. (**c**) Deposition of Au/Ag/Cr nanolayers over the photoresist mask. (**d**) Removal of the photoresist mask via a lift-off process, resulting in a planar array of Au/Ag nanowires with a layered structure. (**e**) Annealing of the Au/Ag nanowires at 800 °C for 2 h to trigger solid state diffusion reaction and form Au–Ag alloy nanowires. (**f**) Formation of nanoporous gold nanowires by dealloying of the nanowires in concentrated nitric acid (~16 M) for 1 h.

The fabrication approach of the planar arrays of nanoporous gold nanowires is presented in Figure 1. First, a silicon substrate is coated with a 200 nm photoresist layer by spin coating (Figure 1a). Then, laser interference lithography is applied to pattern the photoresist mask and create an array of periodic nanograte structures with a height of 200 nm and a pitch of 240 nm, which serve as a mask for metal deposition (Figure 1b). The maskless laser interference lithography technique is capable of patterning uniform and large-area nanopatterns. Wafer-scale and highly ordered nanostructures can be patterned with tunable pattern periodicity and a controllable aspect ratio. After the lithography, Au/Ag/Cr stacked layers are deposited sequentially over the photoresist mask. The 10 nm Cr layer was used to ensure a good adhesion between the Au/Ag stacked layers and the substrate and to avoid material delamination during the removal of the photoresist in a piranha solution in a further stage. The thickness of the Ag and Au layers were varied to control the final composition of the Au–Ag alloy nanowires. After metal deposition, the photoresist mask is removed by a lift-off process in a piranha solution, which leads to the obtainment of an array of metal nanowires with a layered structure (Figure 1d). To form an Au–Ag alloy, the nanowires are subject to an annealing procedure at 800 °C for 2 h (Figure 1e). The obtained nano-alloys are then dealloyed by a free-corrosion process using concentrated nitric acid to leach silver preferentially from the alloy leaving behind a gold skeleton with a sponge-like structure (Figure 1f). The materials and methods in more detail are described in the following section. 

## 3. Materials and Methods

**Large-area nanopatterned template.** The nanopatterned photoresist mask to deposit and form the Au–Ag nanowire arrays were prepared by laser interference lithography with an HeCd laser (325 nm, 50 mW). A negative-tone photoresist (NR7, Futurrex Inc., Franklin, NJ, USA) was spin-coated onto a polished silicon wafer (4 inch) and then exposed with two-beam Lloyd-mirror interferometer [32,33]. The pitch of the nanopatterns can be easily and precisely tuned by adjusting the angle between the two incident laser beams. The photoresist mask pattern employed in this study is an array of nanograted structures (120 nm in width and 200 nm in height) of 240 nm in pitch and an aspect ratio of ~2. The high scalability of the laser interference lithography system allows the nanopatterning over the full area of a 4-inch wafer with 240 nm period nanogratings [33,34,35]. 

**Growth of Au–Ag alloy nanowires and dealloying.** The Au/Ag stacked layers used to create the Au–Ag alloy nanowires were deposited through the photoresist mask by e-beam evaporation (Lesker Inc., Jefferson Hills, PA, USA). Prior to the Au/Ag deposition, a 10 nm Cr adhesion layer was deposited through the mask to ensure a good adhesion and mechanical stability of the Au/Ag stacked layers to the substrate and avoid delamination issues during the lift-off procedure (i.e., removal of the photoresist mask in piranha solution). 

The thickness of gold was tuned between 80 and 120 nm, whereas the thickness of Ag was tuned between 40 and 80 nm. This was achieved by adjusting the depositions times of Au from 400 to 600 s and the deposition time of Ag from 200 to 400 s, allowing for the creation of nanowires with Au-to-Ag thickness ratios between 1 (denoted Condition 1) and 3 (denoted Condition 2). The final thickness of the stacked Au/Ag nanowire was made constant to be 160 nm. Annealing of the Au–Ag nanowires was performed under high vacuum at 800 °C for 2 h to ensure a good mix of the materials and the transformation of the Au/Ag stacked layers into Au–Ag alloy.

The dealloying of the Au–Ag nanoalloys was carried out in 70% concentrated nitric acid (15.7 M, Sigma-Aldrich, St. Louis, MO, USA) at room temperature. When achieving the desired etching time (i.e., 1 h), the samples were then dipped in deionized water for several minutes to stop the dealloying process and remove the residue of nitric acid. As a safety precaution, all the dealloying experiments were carried out inside a fume hood to avoid any possible exposure to nitrogen dioxide (NO_2_), which is a reddish-brown toxic gas emitted as a consequence of the reaction between nitric acid and silver.

**Characterization.** The scanning electron microscope (SEM) micrographs were recorded using a JEOL JSM 7600 F microscope (Akishima, Japan) operating at 5 kV. The chemical compositions of the nanowires were determined by energy dispersive X-ray spectroscopy (EDS) performed on a JEOL 5800LV microscope (Akishima, Japan) operating at 5 kV.

## 4. Results

**Figure 2:**
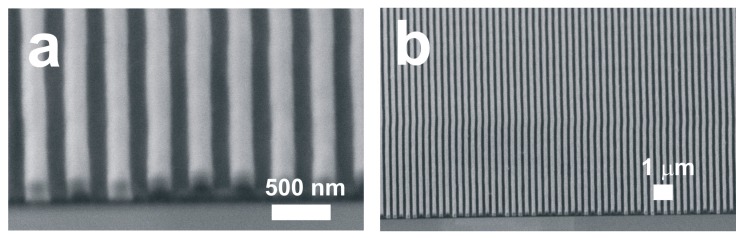
(**a**) High and (**b**) low magnification scanning electron microscope (SEM) images of the nanograted patterns of photoresist with the periodicity of 350 nm.

Figure 2 shows SEM images of the array of nanograting structures of negative photoresist with the period of 350 nm fabricated by the laser interference lithography, corresponding to the schematic shown in Figure 1b. The laser interference lithography allows us to fabricate the nanoscale periodic grating structures uniformly covering a 4-inch silicon wafer. The full wafer-scale nano-periodic patterns could be fabricated within 20 min (UV exposure: ~5 min, baking: ~5 min, and development: ~5 min), due to the great scalability of the interference lithography technique. The interference lithography is an ideal nanomanufacturing method to prepare a large-area nano-template surface for the fabrication of ultra-long nanowires patterning combined with advantages of scalable, fast speed, high throughput, simple control, and low costs. 

**Figure 3:**
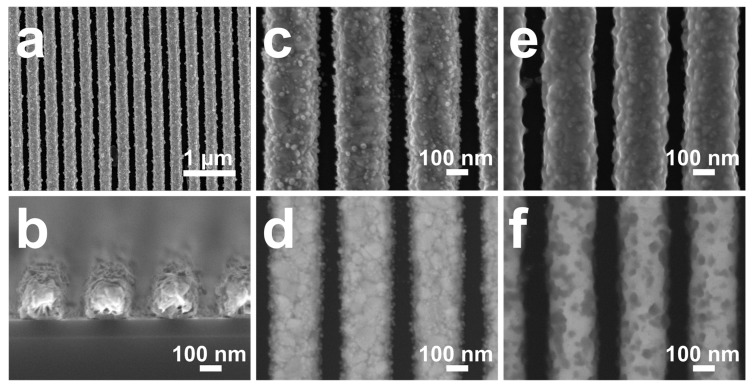
SEM images (**a**,**c**,**e**) top view and (**d**,**f**) backscattered electron top view and (**b**) cross sectional view) of Au/Ag layered nanostructure (**a**–**d**) before and (**e**,**f**) after annealing at 800 °C for 2 h.

It is known that the composition of the alloy strongly impacts the morphology of nanoporous gold obtained by dealloying [29,36]. To control the composition of the Au–Ag alloy nanowires, we varied the ratio between the thicknesses of the Au and the Ag layers, while maintaining the overall thickness of the material constant to be 160 nm. Figure 3a–d shows the SEM images of the stack of Cr (10 nm)/Au (120 nm)/Ag (40 nm) (Au-to-Ag thickness ratio = 3) (Condition 2), before annealing and dealloying, corresponding to the schematic shown in Figure 1d. As can be seen, the nanowires are perfectly organized and exhibit the same alignment of the photoresist lines. Their width is about 250 nm and perfectly matches the width one of the interdistance between two adjacent photoresist lines before the lift-off. Figure 3e,f presents SEM images of Condition 2 after thermal annealing but before dealloying (corresponding to Figure 1e). No significant morphological transformation can be noticed. However, the plan-view SEM images recorded in the backscattered mode clearly show the melting effect of the two metals after annealing, indicating the diffusion of silver within the gold phase. The heterogeneous aspect is an indicator of the presence of a silver gradient from the top of the nanowire toward the core region of the gold phase. 

**Figure 4:**
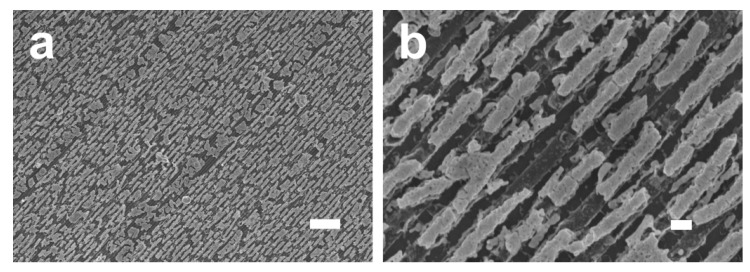
(**a**) Low- and (**b**) high-resolution SEM images of dealloyed Au/Ag nanowires for 1 h in nitric acid. The Au–Ag nanowires were prepared by annealing Au (80 nm) and Ag (80 nm) stacked layers. Scale bars in (**a**,**b**) are 2 µm and 200 nm, respectively.

Figure 4 shows the result of the fabrication of nanoporous gold nanowires after annealing and dealloying of the stack of Au (80 nm)/Ag (80 nm) (thickness ratio = 1) (Condition 1), after following the schematics shown in Figure 1e,f. At this condition, the nanowires were found to break into small pieces (Figure 4). This is attributed to the huge internal stress generated within the alloy during the corrosion process, which results in the generation of cracks within the material [37,38]. In the case of planar nanowires, as the material sticks to the substrate surface, the stress along the wire axis cannot be accommodated, leading to the lateral (perpendicularly to the wire axis) cracking of the nanowires. 

**Figure 5:**
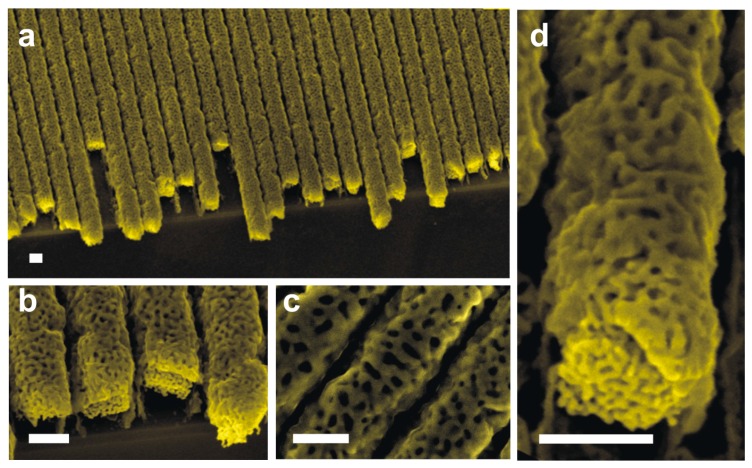
(**a**–**c**) Low- and (**d**) high-resolution SEM images of nanoporous nanowires obtained by dealloying in concentrated nitric acid for 1 h of Au–Ag alloy nanowires prepared by thermal annealing at 800 °C for 2 h of Au (120 nm)/Ag (40 nm) stacked layers. Scale bar in each image indicates 200 nm.

As demonstrated in our previous work, the cracking effect of the material during dealloying due to the huge generated tensile strain generated as a consequence of the removal of silver from the alloy (shrinkage effect) can be overcome by (i) increasing the gold content within the alloy (solution adopted in this work) or (ii) decreasing the nanowires diameter, allowing the material to accommodate the generated strain [29]. We adopted the first solution and aimed to increase the gold content within the Au–Ag alloy nanowires. Figure 5 shows the results of the fabrication of nanoporous gold nanowires annealed and dealloyed with the stack of Au (120 nm)/Ag (40 nm) (thickness ratio = 3). After dealloying for 1 h, unlike the case of the lower thickness ratio of 1, the nanowires made from the higher thickness ratio 3 was not broken (Figure 5a). In addition, the nanowires maintained the regular and parallel organization and showed high porosity (Figure 5b–d). The structure of the material consists of interconnected nanoligaments. Moreover, Figure 5c (plan-view) and Figure 5d (cross-sectional view) show two levels of porosity. The pores at the surface level (Figure 5c) have a size of about 30 ± 5 nm, whereas the one within the bulk of the wire (Figure 5d) has a size of ~8 ± 2 nm. The Au concentrations of the samples before and after thermal annealing were the same (50% of Au for Condition 1 and 75% for Condition 2). With the conditions we used for dealloying, the silver residue within the nanoporous gold remains less than 3%. When examining the cross-section SEM images of the nanowires after dealloying, one can remark the presence of dual porosity within the material: the pores are larger at the top surface region compared to the ones formed within the core region. Such non-uniform pore size along the thickness of the nanowires is probably related to the presence of an Ag concentration gradient generated as a consequence of the annealing process. Indeed, as presented in Figure 3, after annealing, the silver has not uniformly diffused through the gold phase but rather exhibits a gradient concentration with a high amount observed at the top surface region of the nanowires. As the pore size is strongly dependent on the concentration of the less noble metal within the alloy [22], when dealloyed, the regions close to the top surface rich with Ag are found to exhibit a large pore size compared to the core regions where a silver concentration is expected to be much lower. The ligaments at the surface level (Figure 5c) have a width of 34 ± 10 nm, whereas the one within the bulk of the wire (Figure 5d) has a width of ~13 ± 3 nm. The resulting porous nanowires are found to be highly conductive with a sheet resistance as low as 6 Ω/sq. This result is a direct proof for the possibility of using such nanowire arrays for the development of resistive sensors. Indeed, some studies have reported on the use of a single nanowire for the fabrication of sensors with high performances [26]. Due to their superior organization, their high length, and their low sheet resistance, our nanowires with such a planar architecture are expected to significantly increase the sensitivity of this kind of sensor and avoid complicated manipulation procedures for the integration of a single nanowire in the sensor. Moreover, some studies have reported on the high performance of porous gold nanowires when integrated in surface enhanced Raman scattering (SERS)-based sensors in comparison to porous thin films [39,40] due to their shape anisotropy. Our nanowires with such a special architecture can be easily used for the development of such sensors. Moreover, the low inter-distance between adjacent nanowires, the great organization of the features, and the small pore size are expected to strongly enhance the plasmonic effect compared to the metal grating structure [41,42]. Such enhancement in plasmonic properties is an additional parameter that can allow for improvement of the performance of SERS sensors fabricated with such planar nanowires.

## 5. Comparison to Template Technique

**Figure 6:**
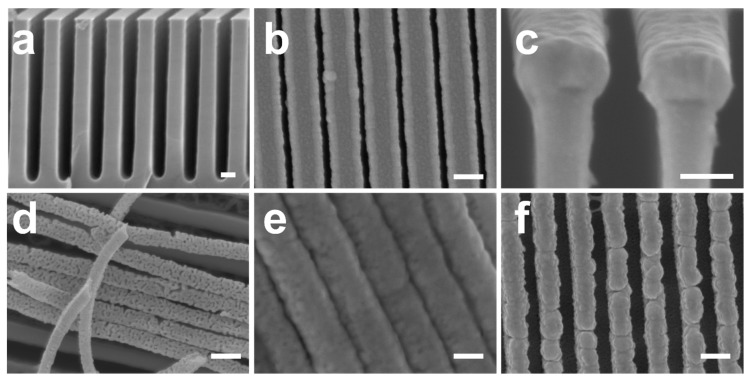
(**a**) Cross-section SEM images of a typical nanopatterned substrate used to grow the Au–Cu nanowires by cosputtering. (**b**) Top-view and (**c**) cross-section SEM images of Au–Cu nanowires with 16 atom % gold and 150 nm in diameters deposited by magnetron cosputtering on nanograted substrate. (**d**–**f**) SEM images of Au–Cu nanowires with different initial gold concentrations after dealloying in concentrated nitric acid (16 M) for 50 s: (**d**) 16 atom %, (**e**) 23 atom %, and (**f**) 26 atom % Au. Scale bar: 100 nm.

In a previous work, we reported on the fabrication of nanoporous gold nanowires by applying electrochemical dealloying to Au–Cu nanowires grown by cosputtering on nanograted substrates serving as a physical template [29]. It should be noted that, prior to electrochemical dealloying, we studied the possibility of fabricating nanoporous nanowires, deposited by cosputtering on nanograted substrates, by free corrosion applied in nitric acid. Figure 6a presents a typical SEM image of the nanograted substrates used as a physical template to grow the Au–Cu nanowires by cosputtering (Figure 6a). Nanowires 150 nm in diameter (Figure 6b,c) and with different gold contents were dipped in concentrated nitric acid (16 M) for 50 s. For nanowires with 16 atom % gold at the initial state (Figure 6d), porosity can be observed, albeit broken and delaminated from the substrate. To overcome the delamination and the cracking effects, we increased the initial gold content within the nanowires. For samples with 23 atom % gold (Figure 6e), while the porosity was found to be very limited (only tiny pores can be observed), the nanowires were found to delaminate from their support and agglomerate together and form a bundle. For nanowires with 26 atom % initial gold content (Figure 6f), a great organization over the substrate was observed after the chemical treatment but no porosity was formed even for long dealloying durations; such an ability of nanowires to resist the nitric acid attack is related to the surface passivation of the nanowire by the gold. To conclude, compared to the work presented in this manuscript, nanowires grown by cosputtering on template substrates suffer from a cracking effect and from delamination issues when dealloyed by free corrosion due to their weak adhesion to the substrate. However, the pore size obtained by dealloying cosputtered nanowires was more homogeneous than the one described in the present study; this is related to the fact that the annealing process applied in this work to the Au/Ag stacked layers does not allow for the formation of a homogenous phase of Au–Ag alloy, as it is the case with cosputtering but rather results in the formation of a concentration gradient within the nanowires.

To overcome the previously mentioned drawbacks encountered when applying the free corrosion process to Au–Cu nanowires grown on nanograted substrates, one can apply dealloying electrochemically. In addition, electrochemical dealloying allows for a better monitoring of the final morphology of the material. However, large-scale manufacturing of nanoporous nanowires as demonstrated in the present manuscript is much easier and practical when performed using the free corrosion process as electrochemical dealloying requires a uniform current injection throughout the sample, which is very challenging for large-scale manufacturing. On the other hand, free corrosion has already proved its ability of creating nanoporous gold on large scale [43].

## 6. Conclusions

It is successfully demonstrated here that large-area (wafer-scale) planar arrays of nanoporous gold nanowires can be conveniently achieved by dealloying Au–Ag alloy nanowires pre-patterned on a nanostructured template substrate prepared by laser interference lithography and lift-off processes. It shows that the lift-off process is also as effective as the method using a shadowing effect in forming well-organized metal alloy nanowires. The results show that the thickness ratio between the top Au layer and the underlying Ag layer of the nanowire alloy plays an important role in achieving the uniform and well-regulated nanoporous gold nanowires with good porosity. The relatively low thickness ratio (e.g., around 1) results in cracks during the dealloying process, which is mainly due to the internal residual stress generated during dealloying. However, the relatively high thickness ratio (e.g., around 3) mitigates such residual stress issues and enables us to obtain well-organized porous gold nanowires with little crack or breakage. Such planar arrays of large-area well-organized porous gold nanowires are of great importance in many applications, such as SERS sensing of contaminants such as heavy metals in water, actuators, catalysis, and optical polarizers.
